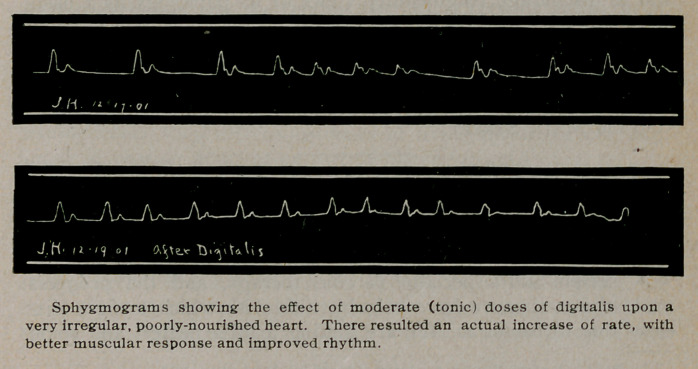# Heart Nutrition1Read before the Section on Medicine, Buffalo Academy of Medicine, October 11, 1910.

**Published:** 1911-03

**Authors:** Eli H. Long

**Affiliations:** Buffalo, N. Y. 1335 Main Street


BUFFALO MEDICAL JOURNAL

VOL. LXVI. MARCH, 1911. No. 8

### ORIGINAL COMMUNICATIONS

### Heart Nutrition[1]

BY ELI H. LONG, M.D.,
Buffalo, N. Y.

THE importance of this topic rests upon the fact that the great majority of cases of cardiac disease that we are called upon to treat are essentially myocardial. Whether the point of departure in the pathology of a case be endocardial and valvular, or arterial in the nature of sclerosis, or be it a primary cardiac degeneration, when the case presents for treatment we have a myocardium that is unequal to the task imposed upon it and the question of its nutrition in order to betterment becomes the primary question. In discussing the topic, the writer's aim is simply to emphasize some essential points. No attempt will be made to treat all phases or to discuss many remedial measures.

As a basis of study it is proper to review the main points in the physiology of the heart. A hollow muscular organ, its structure and function are complex, providing for the successive filling and emptying of its four chambers in order to the propulsion of two streams of blood to maintain the lesser pulmonary and the greater systemic circulations. Corresponding to the force required in each of these, the left side of the heart is stronger than the right. Being an involuntary muscle, its contraction is independent of the cerebro-spinal or general sympathetic nervous system. This point will bear emphasis in statement of the fact that the power of rhythmic motion of the heart resides within its own structure. Any nerve influence from without has nothing to do with originating or maintaining its action, but can only modify its rapidity or force.

Accessory to the heart structures proper we recognise that the dilatable arch of the aorta possesses something of a reservoir function, being capable of accommodating an excess of blood temporarily; also the distensible and contractile arterial tree contributes the elastic force that converts the intermittent flow from the heart into a continuous supply to the capillaries. As will be seen, these factors are related not only to the distributive function

---

1. Read before the Section on Medicine, Buffalo Academy of Medicine, October 11, 1910.

of the heart but to its nutrition as well. The extrinsic cardiac nerves are the vagus, which is the inhibitory or depressor nerve, and the sympathetic fibers, which contribute the accelerator influence. Concerning the origin and conduction of the impulses that result in auricular and ventricular contractions at regular intervals, much work has recently been done and important knowledge has been gained. Briefly, it is now recognised that the impulse normally starts about the mouth of the superior vena cava and travels downward, first inducing contraction of the auricles, then through the auriculo-ventricular bundle it is distributed by the divisions of the latter to both ventricles.

We need not be greatly concerned with the disputed question whether the impulse is myogenic or neurogenic except to note that it has been demonstrated that the impulse need not always follow the usual course, but may originate in various parts of the heart. That is, with the conducting bundle ligatured between sinus and auricles and between auricles and ventricles, it has been found that auricles and ventricles may take on independent action. This may be taken to support the myogenic theory that the impulse to contraction arises in the heart muscle. Gaskell assumes that the muscle fiber secretes a substance that is capable of stimulating its contraction.

The circulatory relations of the heart are unique in that it has the most abundant blood supply of any muscle, and its arteries of nourishment, the coronaries, being the first to be given off from the aorta, receive the benefit of the full force of the arterial current in its rebound from the closure of the aortic valves. The blood supply is sufficient to provide for growth of the organ when necessary.

In the management of diseases of the heart we come to recognise that the real indications for treatment do not arise from any exact lesion of the valves, for valvular defects of almost any kind may exist without palpable disturbance of the circulation; while on the other hand, most serious conditions of the heart structure often exist without any discoverable lesion. The indications for treatment must be found in the condition of the circulation as a whole and the question that centers in the heart is whether it is doing, or is capable of doing, the work demanded of it. We cannot repair a leaky valve or dilate a narrowed orifice, but in nearly every case we can do much to improve and maintain the nutrition of the organ, enabling it to compensate a defect and to work under improved conditions.

We need pay little attention to the innervation of the heart, except to remove toxic elements that may be affecting rhythm or strength of action. Trophic nerve influence is too indefinite a factor to furnish any basis of treatment. These considerations lead to the proposition that the factor of primary importance to heart function is *good muscular response.* This depends upon a normal condition of its muscular tissue and a proper supply of good blood, that is to say, normal structure plus good nutrition. Where either of these fail we may have the heart's function seriously imperiled. Toxic states of the blood, as in acute infectious diseases, must often be regarded as the direct cause of death, while in more chronic conditions where elimination is constantly deficient, a lesser degree of toxicity of the blood must have an influence in inducing degeneration and preventing proper nutrition of the heart. On the other hand unsuspected degeneration, from one cause or another, is often present in middle and advanced life and must be held responsible for the sudden deaths that occur in apparently well men in the midst of an active business life. These factors are the less appreciated because they are less apparent through our ordinary means of examination.

Its importance granted, how is the muscular response of the heart to be measured? Practically by adding to its work and noting its reaction to meet the demand. A heart with good response will react by greater frequency of the pulse. Gaskill analyses this factor of muscular response, recognising (1) stimulus production, (2) excitability, (3) conductivity, (4) contractility. and (5) tonicity, as distinct functions.[1] It can readily be appreciated how a weak link in this chain of functions can give various and obscure disturbances of the myocardium; likewise how a break in the same may abolish the power of response and cause sudden death. All things being equal, the readiness of response and the regularity of rhythm should give a fair indication of the state of the myocardium. And a heart whose action easily becomes quickened upon slight exertion must be rated better than one whose action remains continuously slow in spite of exertion.

A second factor related to heart nutrition is that of *work,* or the ratio between the heart's strength and its work. Cardiac disability is a relative condition, usually due to disturbance of this ratio by continuously demanding of a weakened or defective heart work that is beyond its strength. This may be caused by a sudden strain of the heart muscle by extraordinary exertion, or by its effort at maintaining the circulation with disabled valves or a weakened myocardium. Whenever valvular disease leads to circulatory failure or whenever a previously established compensation becomes broken, the factor of overwork is present and of primary importance. The heart cannot get away from its task, but rather with continued disability its work increases. It does its best under the conditions by working faster to make up for its lack of strength, and we are often amazed at the resources

1. Schaefer's Textbook of Physiology.

which it displays in doubling its rate for days before giving up the struggle.

There is one constant result of a heart working against too great a load, that is stagnation of blood at some point. Ordinarily stagnation occurs in the periphery because the pressure there is lower and the heart does not give enough force to the blood stream to return it in full. The result is edema. If the heart is strong, with its work increased by the high blood pressure of arterial sclerosis, the stagnation may take the form of aneurism at some point of weakness in the arterial wall. If the increased work is due to obstruction in the pulmonary circulation, or at the mitral orifice, stagnation will be upon the venous side, and the liver, kidneys and abdominal vessels will be engorged, But the place where stagnation is well nigh invariably present in any and all forms of cardiac disease is in the heart itself. The dilatability of its chambers is taken advantage of and often away beyond the limit of the elasticity of their walls, so that they become incapable of properly expelling their contents. This stagnation of blood within the chambers in itself becomes a serious hindrance to the heart's function. Distension means a stretching and thinning of the muscular wall and in the same degree an accumulation of blood, which the weakened walls are expected to contract upon It is plain that the greater the distension the more work the muscle is called upon to do and the greater the disadvantage under which it must work. By the same conditions the tonicity of the muscle must be lessened, so that extreme relaxation more readily occurs. This condition of *dilatation* constitutes a third factor of importance in the study of these cases.

The management of heart nutrition, then, involves chiefly the maintenance of good muscular response, the suiting of the heart's work to its strength and the relief of dilatation. The remedies may be discussed in relation to all three factors at once. Most cases that present for treatment fall into either of two classes, first, valvular cases that lack compensation but have a fair condition of heart muscle, with good response on its part, occurring mostly in young people, and second, later cases that show some degeneration of structure as evidenced by irregularity or deficient muscular response. The former usually respond well to proper treatment, though much can be done also for the latter, even to securing good heart action for years. Age is a telling factor in prognosis because of the natural tendency toward degeneration with advancing years; and this is dependent at least as much upon arterial conditions as upon cardiac. The attention given in recent years to diseases of the arteries has taught us that circulatory conditions cannot be properly treated by attention to the heart alone, but that the circulation must be studied and treated as a whole.

Without question the first measure to be considered in treatment is rest. Absolute rest of the heart being impossible, we have before us the task of adapting the work of the muscle to its strength so as to secure relative rest. The view taken here is, that when heart disability occurs it is not simply a physical disability due to an imperfect valve, or a distended ventricle, but that the failure of proper nutrition of the organ is an equally present factor and one that is usually capable of being remedied. Our aim in securing relative rest, therefore, is to put the heart into a condition where the gain in nutrition exceeds the outlay in work. It is not first a matter of giving cardiac stimulants, for they may not be needed; and it is better to obtain our estimate of the resources of the heart without them. The recumbent posture strictly enforced, meanwhile forcing elimination by the bowel, will enable us in a few days to see how well the heart is able to maintain the circulation upon the level; then we can judge of its further needs.

If symptoms of embarrassment disappear medicine may be unnecessary; but if the symptoms show no improvement digitalis is indicated for the time being, as much for its indirect influence in improving heart nutrition as for its direct stimulant action. It is particularly where much dilatation exists that this drug is required. But we should employ other means of lessening the heart's load. Particularly in the second class of cases, later in age or in clinical history, can we make use of the now well-established measures of removing peripheral resistance in the circulation by dilatation of the cutaneous arterioles. This is accomplished by means of the warm brine bath, the effervescent Schott bath, or by massage and surface friction, and in some cases by the use of the nitrites. Preference should be given to those measures that secure dilatation without depressing the tonicity of the muscular tissue; therefore, the nitrites have gradually come to take a place of secondary importance in most cases because their action is essentially depressant.

There are occasional cases in which sleeplessness is so marked a factor as to prevent rest. Here it may be advisable to resort temporarily to nerve depressants and even to morphine, if other measures do not confer the ability to sleep. The importance of digitalis in some cardiac cases renders it advisable to know just what may be expected from this drug and what the plain indications are for its use. A brief review of its action, therefore, is here proper. Taking a preparation that fully represents the drug, e. g., the tincture, it is recognised that it has a complex action embracing three distinct factors. The first, best and most desirable action is upon the heart muscle direct, increasing its tonicity and inducing more powerful contractions. A second action is upon the vagus centers in the medulla, by stimulating which the heart's action is somewhat slowed and depressed, giving the same effect as does aconite. Thus we have an antagonism between the first and second factors in the action of digitalis, between muscular action and inhibition, the result usually being a stronger and somewhat slower action. A third factor is that of vasoconstriction due to direct action upon the muscular walls of the arterioles and probably also to stimulation of vasomotor centers in the medulla. This diminishes the flow of blood into the capillaries and increases the peripheral resistance to the heart's action, causing with the first factor a rise of arterial blood pressure. The diuretic action of the drug is mainly incidental to the rise of blood pressure.

It must be asserted that this drug has been, and still is, used too much and without proper discrimination. We shall do well to regard it as an emergency drug, to be used in obedience to clear indications and never simply because a certain lesion is present. A drug that forces a weak heart to more powerful action and at the same time adds to its work by increasing the peripheral resistance must possess counterbalancing influences to give it value; and these are found in its power to slow the action, giving more rest between contractions, and in the positive diminution of a dilated heart by the increase of tonicity and the more powerful contractions. In fact, its value lies largely in those factors of its action that contribute to its better nutrition, namely, rest, contraction of its volume, by which not only is dilatation lessened but the venous blood is more completely squeezed out of its tissues to be replaced by fresh blood, and its more abundant blood supply through rise of blood pressure in the aorta. Mackenzie[1] places emphasis upon its power to increase tonicity of the heart muscle, by which he means the function that antagonises relaxation, so that the ventricular contractions are not only firmer but longer maintained, and relaxation during diastole is lessened.

The clear indication for digitalis in stimulant doses is the presence of dilatation to a disabling degree, with the heart muscle in a fair condition. The greater the extent of degeneration, the less can be expected from this drug, though the time arrives in nearly all cardiac cases when its use is resorted to with more or less benefit. In older cases, with the arteries less elastic and their walls thickened and rigid, the vasoconstrictor action of the drug may be a disadvantage, adding to the resistance against which the heart is working and possibly reducing the amount of blood flowing into the capillary areas. Here we may still often secure excellent results from digitalis by combining it with a vasodilator. In fact, the freer supply of blood thus secured to the

1. Mackenzie, Diseases of the Heart.

capillaries, including those of the heart, by opening up the arterioles, with the increased arterial force from the heart, fulfills the first condition of a better nutrition, which the heart badly needs.

Sphygmograms showing the result of combined use of digitalis and nitroglycerin in a person aged 61, with heart somewhat irregular.

While the above remarks apply to the use of digitalis in doses sufficiently large to call forth its full action to the point of slowing the pulse perceptibly, I am convinced that we may also see benefit from smaller doses that do not force the heart's action and that do not always slow it,—a tonic action that contributes to a better

Sphygmograms showing the effect of moderate (tonic) doses of digitalis upon a very irregular, poorly-nourished heart. There resulted an actual increase of rate, with better muscular response and improved rhythm.

tone and a better response. A tracing that illustrates this action shows an irregular slow pulse actually increased in frequency, while becoming more regular, under the drug. Remembering that, as a stimulant, digitalis is an emergency drug, we may still want its tonic action by continued smaller doses for some time after the emergency period has passed.

A good blood being the basis of good nutrition, we must supply to the heart a blood that is not only nourishing, but that is non-toxic. To this end proper elimination must be maintained. This often requires the forcing of bowel elimination particularly. We may assume, when the circulation is insufficient so that oxidation is lessened. that the elaboration of cell products is imperfect and that substances more or less deleterious accumulate in the tissues and in the blood. These, circulating within the heart's structure, must interfere with its nutrition; and we cannot get very far in improving our cases without ridding the blood of such material by free elimination.

Ultimate improvement will be aided by employing another means of promoting nutrition,—exercise. As applied to the heart, exercise must be graded exactly to meet the purpose of stimulating hypertrophy, recognising the principle that, to secure such growth of its muscle, the work of the heart must be gradually increased so as to maintain a stimulating degree of tension of its walls. In cases of lost compensation, exercise must be at first tentative and slight. Probably massage or passive movement of extremities will be all that the heart can stand. From this we can gradually go to active, then to resistive movements, even before the patient can sit up. From that point on ordinary means of exercise may follow, with proper supervision.

In conclusion, it is hoped by the writer that these unoriginal thoughts may serve to emphasise the unity of cardiac structures and to discourage fragmentary views of its pathology as a basis for treatment; that the importance of its nutrition may be fully appreciated and that our therapeutic measures may be promptly addressed to conserving the same as the foundation of real improvement in its disease.

1335 Main Street.

## Figures and Tables

**Figure f1:**
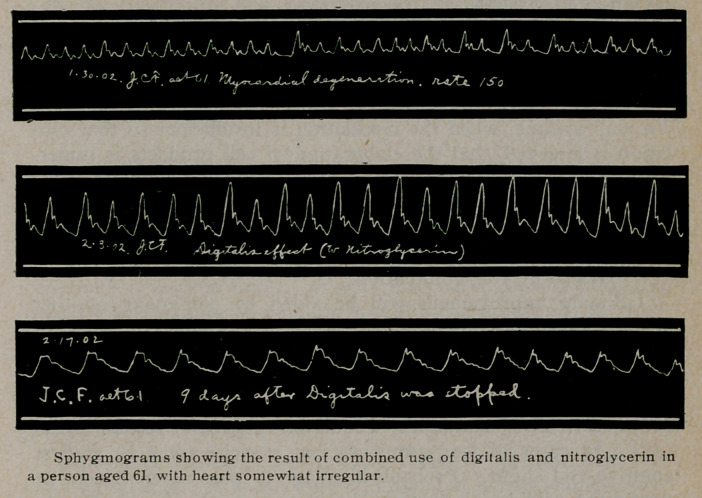


**Figure f2:**